# Small-scale field evaluation of the efficacy and residual effect of Fludora^®^ Fusion (mixture of clothianidin and deltamethrin) against susceptible and resistant *Anopheles gambiae* populations from Benin, West Africa

**DOI:** 10.1186/s12936-018-2633-6

**Published:** 2018-12-29

**Authors:** Fiacre R. Agossa, Gil G. Padonou, Arsene Jacques Y. H. Fassinou, Esdras M. Odjo, Osei K. Akuoko, Albert Salako, Zinsou C. Koukpo, Udoka C. Nwangwu, Bruno Akinro, Michel Sezonlin, Martin C. Akogbeto

**Affiliations:** 1 Centre de Recherche Entomologique de Cotonou (CREC), 06BP2604 Cotonou, Bénin; 2 Laboratoire Evolution, Biodiversité des Arthropodes et Assainissement, FAST-UAC, Abomey-Calavi, Bénin; 3 Ecole Doctorale Sciences de la Vie et de la Terre, FAST-UAC, Abomey-Calavi, Bénin; 4 Noguchi Memorial Institute for Medical Research University, Accra, Ghana; 5 National Arbovirus and Vectors Research Centre (NAVRC), Enugu, Nigeria

**Keywords:** Fludora^®^ Fusion, Clothianidin, Deltamethrin *Anopheles gambiae*, Efficacy, Benin

## Abstract

**Background:**

In recognition of the threat of insecticide resistance in vectors of malaria, the WHO Global Malaria Programme recommends the development of an appropriate and comprehensive response to insecticide resistance. In principle, good resistance management practice requires the application of multiple insecticides of different modes of action, for example, in rotations and mixtures. Insecticides recommended by the World Health Organization for indoor residual spraying and long-lasting insecticide nets are limited. It is, therefore, judicious to prevent the rapid spread of insecticide resistance by evaluating new insecticides formulations with different modes of action and long residual effect.

**Methods:**

Fludora^®^ Fusion, a new neonicotinoid IRS formulation (a mixture of 500 g/kg clothianidin and 62.5 g/kg deltamethrin applied 200 mg ai/sqm + 25 mg ai/sqm, respectively) was tested. Small scale field evaluation of this product was conducted in the district of Dangbo in Benin, to compare its efficacy and residual effect on cement and mud walls against those of clothianidin 200 mg ai/sqm (WG 70) alone, and of deltamethrin 25 mg ai/sqm (WG 250) alone. WHO wall cone bioassays were conducted monthly with laboratory susceptible Anopheles “Kisumu” and wild *Anopheles gambiae* sensu stricto (s.s.) population from Dangbo. The induced mortality by each treatment per wall substrate for 24 h, 48 h, and 72 h post exposure were recorded every month and analysed.

**Results:**

Fludora^®^ Fusion and clothianidin WG 70 showed mortality rates over 80% WHO bio-efficacy threshold on cement walls either with susceptible or resistant *An. gambiae* s.s. over a period of 10 and 9 months, respectively. Treatment with Fludora^®^ Fusion and clothianidin WG 70 on the mud walls showed residual effect for 6 months and 5 months respectively against both susceptible and resistant mosquitoes. During the whole evaluation period, deltamethrin WG 250 showed mortality rates below 80% against resistant Anopheles population. Furthermore, the knock down rates observed with the Fludora^®^ Fusion combination were significantly higher (p < 5%) than those induced by Clothiandin WG 70.

**Conclusion:**

Both the Fludora^®^ Fusion combination and clothianidin alone showed very good and lasting efficacy for IRS against resistant Anopheles with some residual benefit provided by the combination. The residual efficacy of the Fludora^®^ Fusion combination evaluated at 10 months shows this product is a good candidate for IRS interventions.

**Electronic supplementary material:**

The online version of this article (10.1186/s12936-018-2633-6) contains supplementary material, which is available to authorized users.

## Background

Over the last decades, the scale up of distribution of long-lasting insecticidal nets (LLINs) and indoor residual spraying (IRS) with insecticides to combat malaria vectors, as well as other interventions directed in improving malaria diagnosis and treatment, have contributed to the reduction of the mortality and morbidity linked to malaria across sub-Saharan Africa [1]. Most of the National Malaria Control Programmes (NMCP) have based their malaria vector control on the use of insecticides. The successes noted are threatened by the increase in insecticide resistance in Anopheline malaria vectors [2–5]. Malaria vectors from Tiassalé (Ivory Coast) have developed resistance to all four of the classes of insecticides recommended by the World Health Organization (WHO) [6]. If the current trends of insecticide resistance continue unabated, the gains made though vector control will be lost. Looking forward to the new generation of insecticides on the market, the solution proposed by the national and international community is to optimize the efficacy of vector control by the management of resistance to the existing insecticides.

In Benin, the NMCP has recommended non-pyrethroids for IRS implementation with a view to reducing pyrethroid resistance which is widespread in the country [5]. Indeed, the NMCP started IRS implementation with bendiocarb (a carbamate) from 2009 in the Atacora area. Within 4 years, vectors showed resistance to this insecticide [4]. This situation prompted the programme to implement a mosaic pattern of IRS with both bendiocarb and pirimiphos-methyl (an organophosphate) in 2013 and from 2014 to today with pirimiphos-methyl only. The monitoring and evaluation of IRS activities has shown that both insecticides are very effective. However, their residual effects are very short (under 6 months) and the cost of pirimiphos methyl is 4 to 8 times the cost of pyrethroids, limiting the ability of NMCPs to spray a big area and sustain IRS programmes [7]. Research to identify effective long-lasting insecticides for which the cost is acceptable and which have a residual effect of at least 6 months is necessary.

The Global Plan for Insecticide Resistance Management (GPIRM) from the WHO Global Malaria Programme, calls for the use of alternate insecticides to pyrethroids in order to manage pyrethroid resistance, but this is limited by the limited availability of alternative insecticides and cross resistance between some of them. The best solution to mitigate the spread of vector resistance is to avoid subjecting mosquitoes to the same mode of action over an extended period in order to limit the selection pressure for resistance genes developed by malaria vectors. This is why the Roll Back Malaria/WHO and the Vector Control Working Group have recommended the use of different insecticides, with different modes of action, in rotation or in mosaic for resistance management. However, there are not enough insecticides with different mode of action which are recommended by the WHO to easily achieve this goal. The NMCPs are currently facing the problem of choice of the best strategy to control resistant mosquitoes. The Benin NMCP has, therefore, decided to look for long-lasting effective insecticides with new modes of action by evaluating their efficacy in the national epidemiological context against local resistant vectors. Sustainable and rational deployment of effective malaria vector control tools in insecticide resistance management programs will therefore save lives and improve health. Fludora^®^ Fusion is a mixture formulation developed by Bayer CropScience for IRS which contains the neonicotinoid, clothianidin and pyrethroid, detamethrin.

It is expected that the mosquito population resistant to deltamethrin will be killed by clothianidin and the presence of two modes of action should, in theory, slow down the development of resistance to the new compound. This study was implemented to access the efficacy and the residual effect of Fludora^®^ Fusion in natural conditions against resistant and susceptible *Anopheles gambiae* sensu stricto (s.s.). The result of this study will show the potential of Fludora^®^ Fusion to contribute to the resolution of the problem faced by the NMCPs for sustainable and cost-effective IRS solutions.

## Methods

### Study site

The study was carried in Dangbo located about 60 km from Cotonou. At the last demographic census in 2013, Dangbo’s population was estimated at 88,409 inhabitants (with 14,473 households) of which 46.34% were under 15 years old [8]. The climate is subequatorial with annual rainfall of 1200.83 mm. The average annual temperature is 26.5 °C. The relative humidity throughout the year fluctuates between 70 and 94%. The vegetation of the locality is generally made of savanna where palm trees predominate (natural or planted). There are two rainy seasons (the major starts from April to July and the minor one from September to November) and two dry seasons (August to September and December to March). In 2012, the prevalence of clinically diagnosed and confirmed malaria cases was 9.13%. Dangbo is characterized by two different reliefs: a low-altitude plateau and a marshy plain. The marshland is used for vegetable farming [8]. Land management in the agricultural area creates suitable larval habitats for *An. gambiae* s.s., the main malaria vector [9]. Two types of wall substratum are mostly encountered in Dangbo; theses are cement and mud “banco”. These two types of substratum were considered in this study.

### Mosquitoes tested

The laboratory susceptible strain “Kisumu” and the wild population *An. gambiae* sensu lato (s.l.) from the study area (Dangbo) were used. The larvae of the wild *An. gambiae* s.l. were collected monthly from the field in the district of Dangbo and reared to adult stage at “Centre de Recherche Entomologique de Cotonou (CREC). The mosquitoes aged from 2 to 5 days were used monthly for cone bioassay, for WHO tube tests and for the characterization of the resistance mechanisms exhibited by the wild population (KDR and detoxification enzymes).

### Insecticide formulations evaluated

- Fludora^®^ Fusion is a product (insecticide) developed by Bayer for Indoor Residual Spraying. The formulation type is a Wettable Powder (WP) in water soluble bags (WSB) (available in 100 g sachets). The product contains a mixture of two active ingredients: 500 g/kg clothianidin + 62.5 g/kg deltamethrin. The application rate of the product is 200 mg clothianidin/sqm and 25 mg deltamethrin/sqm.
- K-Othrine WG 250: containing 250 g/kg deltamethrin applied at 25 mg/sqm.
- Clothianidin WG70: containing 700 g/kg clothianidin applied at 200 mg/sqm.

These formulations were tested to see if pyrethroids resistance can be managed with the new insecticide, clothianidin which has a different mode of action or whether the efficacy of pyrethroids can be reinforced with clothianidin against resistant mosquitoes.

### Selection of participant households, informed consent and insecticide treatment design

After receiving authorization from National Ethical Committee for Health Research (CNERS) to implement the study, heads of households were consulted for permission to spray their dwellings. Only households who gave their consent were selected. The selected houses were those of adults, leaders of households, consenting for the study. They were informed on the rationale and methods of the study, the potential side effects of insecticides, the benefits of the study, the confidentiality related to the study, the contact addresses of the investigators of the study, their right to refuse or discontinue the study. In order to avoid the potential risk of the side effects of IRS, or spillage of insecticide outside dwellings and contamination of the phreatic layer, we excluded local people with allergies, those whose walls are cracked and those of wetland and marshy areas. Also, to avoid the contamination of control houses (unsprayed), they were located at least 2 km away from sprayed houses.

**Fig. 1:**
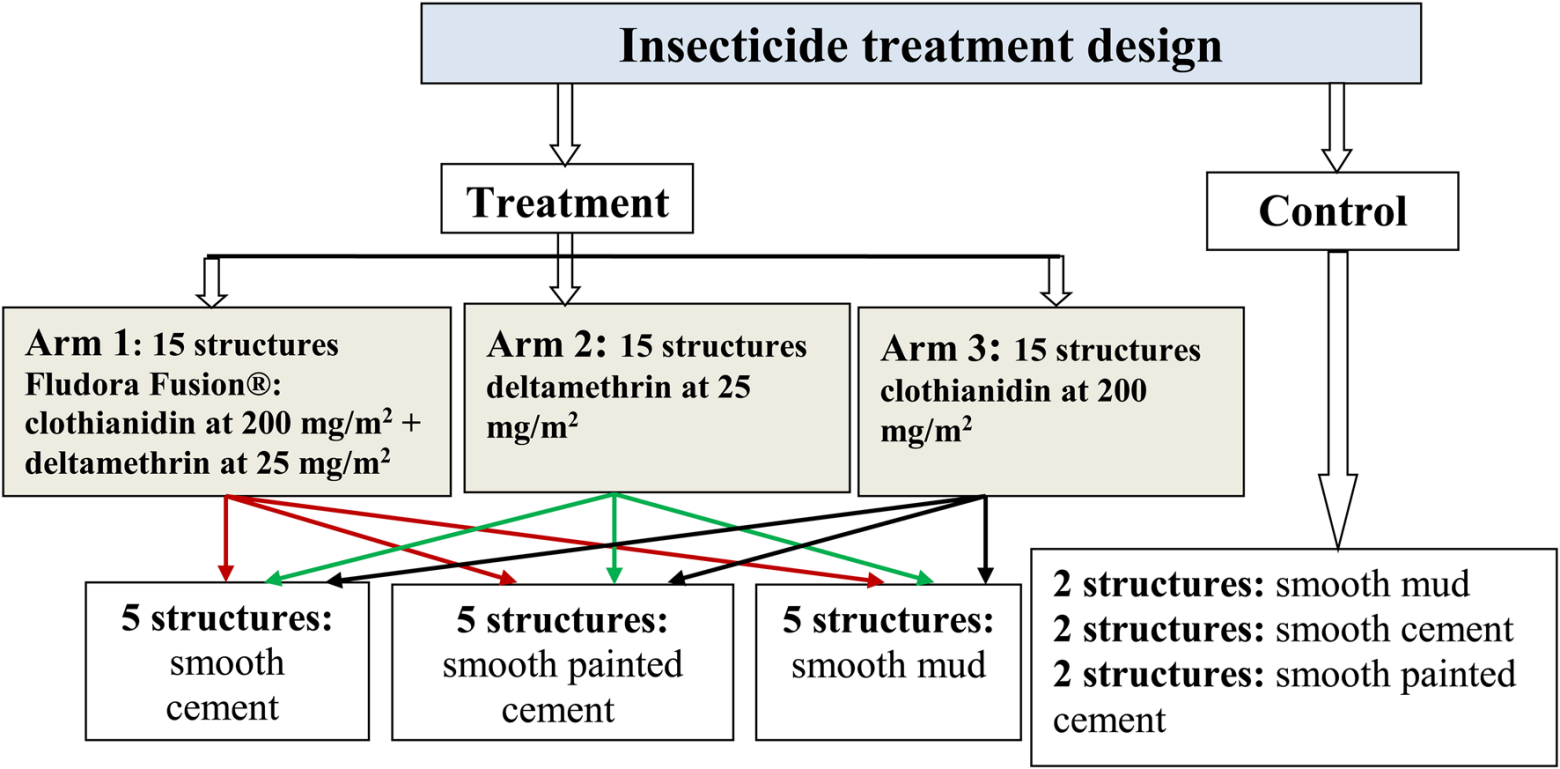
Insecticide treatment design

In order to obtain a good number of replicates needed for statistical analysis at the end of the trial, at least, 51 structures were selected as indicated below (Fig. 1).

### Preparation of insecticide solution and spraying

The preparation of insecticide solutions were made on site before departure to houses to be sprayed. Standard nozzles recommended by the WHO in the fight against malaria vectors were those of 8002 or 8001 type. With 8002 nozzle (for porous mud walls) a volume of 40 mL insecticide solution per minute per m^2^ was projected and 20 mL of the insecticide solution per minute per m^2^ was also sprayed with 8001 nozzle (for non porous cement walls).

Therefore:

#### Porous wall (mud walls)

1. Deltamethrin WG250—dose rate of 25 mg/m^2^: Q = (10,000 mL × 25 mg)/40 mL = 6250 mg for 10L of water were needed In 25 g (25,000 mg) bottle of deltamethrin formulation at 25% active ingredient: 25,000 mg/4 = 6250 mg. 25 g (1 bottle) of deltamethrin formulation were diluted in 10 L of water.
2. Clothianidin WG700—dose rate of 200 mg/m^2^: 70 g (1 bottle) of clothianidin formulation were diluted in 10 L of water.
3. Mixture of clothianidin 200 mg/m^2^ + deltamethrin 25 mg/m^2^: 100 g (1 bottle) of mixture clothianidin 200 mg/m^2^ + deltamethrin formulation were diluted in 10 L of water.

#### Non porous wall (cement walls)

1. Deltamethrin 25 mg/m^2^: Q = (10,000 mL × 25 mg)/20 mL = 12500 mg for 10 L water were needed. In 25 g (25,000 mg) bottle of deltamethrin formulation at 25% active ingredient: 25,000 mg/4 = 6250 mg. So 50 g (2 bottles) of deltamethrin formulation were diluted in 10 L of water.
2. Clothianidin 200 mg/m^2^: 140 g (2 bottles) of clothianidin formulation were diluted in 10 L of water.
3. Mixture clothianidin 200 mg/m^2^ + deltamethrin 25 mg/m^2^: 200 g (2 bottles) of mixture clothianidin 200 mg/m^2^ + deltamethrin formulation were diluted in 10 L of water.

The maximum safety instructions and protective measures were observed during the spraying. The operators who treated the walls wore the requisite protective clothing: cover all or long-sleeved shirt and trousers, hat, rubber boots, gloves and particle-filtering half-mask.

### Measurement of pH of the wall substrates

The treated structures were old, however, the pH of cement and mud plastered walls were tested a day before spraying. This was done by using a scalpel to scrape a small quantity (5 g of substrates) from the wall surface into a petri dish. The substrates were dissolved in distilled water, and then the pH meter was submerged into the concrete or mud solution for reading.

### Status of resistance of the wild *Anopheles gambiae* s.s. population exposed to treated walls

Susceptibility tests using WHO tubes were performed by using adult mosquitoes aged 2–5 days. Detection of Leu–Phe *kdr* mutation was performed by PCR from genomic DNA following the protocol of [10]. Activity of detoxification enzymes was also analysed. Before exposing the wild populations to treated walls, the mechanisms involved in the resistance were identified to evaluate its response in contact with the mixture; clothianidin + deltamethrin (Fludora^®^ Fusion) and clothianidin.

### Bioassays

A laboratory colony *An. gambiae* Kisumu which is fully susceptible to all insecticides and a wild population of *An. gambiae* s.s. resistant to insecticides collected around Dangbo were used for the bioassays. The WHO cone bioassays [1] were carried out in the first week then every month till the eleventh month post treatment. Four cones were placed on each wall at different levels (500 cm, 1 m, 1.5 m and 2 m). Ten unfed (2–5 days) old female *Anopheles* mosquitoes were exposed for 30 min in each cone. The bioassays were conducted each month at the same position of each level over 11 months of the study duration. Mosquito knock-down was recorded at 30 min as well as mortality rate after 24, 48 and 72-h post-exposure. Mosquitoes exposed to unsprayed substrates were used as controls. When the control mortality is between 5 and 20%, corrected mortality is determined using Abbot’s formula. If mortality in controls exceeds 20%, the whole test has been discarded and repeated.

### Statistical analysis

The raw data was entered and managed in an excel database. The statistical analyses, as the logistic regressions, the calculation of the confidence intervals, the significance tests and the graphs are made with the R software (http://www.r-project.com/).

## Results

### Analysis of pH of substrates walls

The pH of smooth cement walls ranged from 9 to 10 (mean pH = 9.3) which were all over 7; the pH of smooth painted cement walls ranged from 9 to 10 (mean pH = 9.2) which were also over 7 and the pH of smooth mud walls ranged from 5 to 8 but the mean pH was under 7 (Table 1).

**Table 1:** pH values of wall substrates used in the study

Wall type	Structure ID	Respective pH values	Mean pH	Standard deviation
Smooth cement wall	SC1–SC15	10; 9; 9; 9; 10; 10; 10; 10; 9; 9; 9; 9; 9; 9	9.3	0.48
Smooth painted cement walls	SPC1–SPC15	9; 9; 9; 9; 9; 9; 9; 9; 9; 9; 10; 10; 9; 10; 9; 9	9.2	0.41
Smooth mud walls	SM1–SM15	6; 6; 8; 6; 5; 5; 6; 6; 6; 6; 8; 6; 8; 6; 6	6.2	1.12

### Susceptibility of wild population of *Anopheles gambiae* to insecticides

**Fig. 2:**
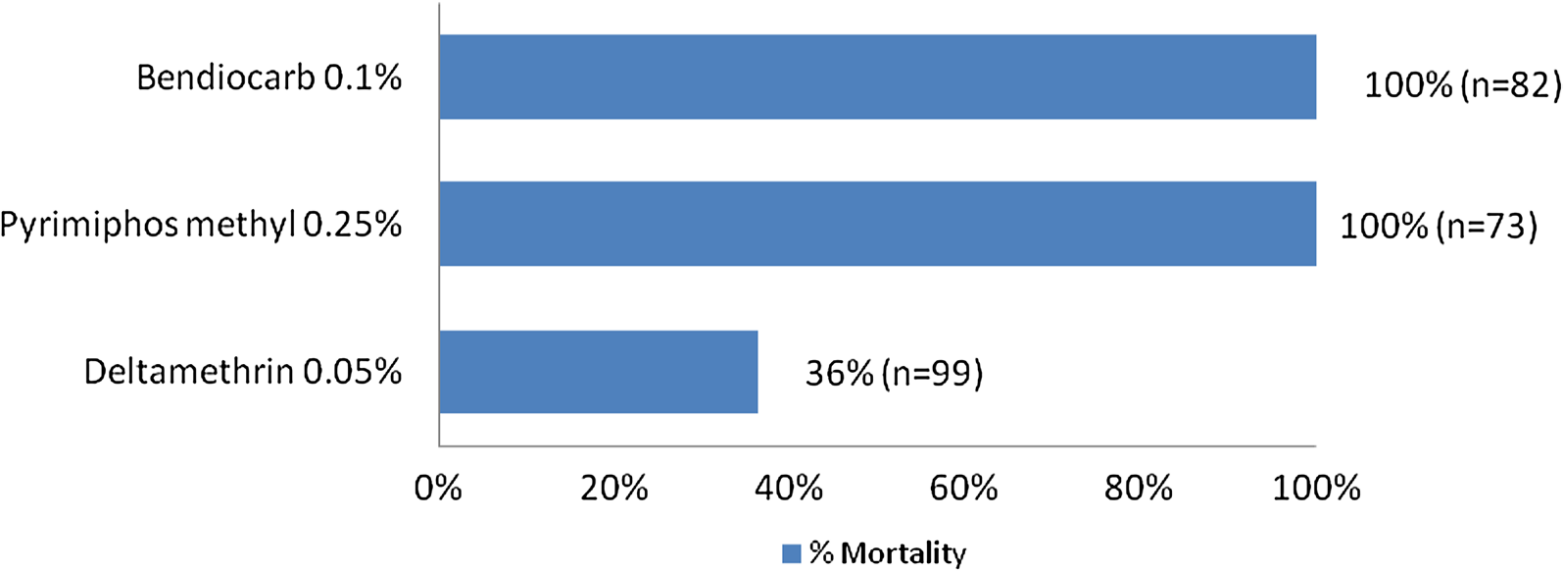
Percentage of mortality of adult *Anopheles gambiae* s.s. populations from Dangbo to pyrethroid, carbamate and organophosphate

Results of susceptibility tests showed that wild population of *An. gambiae* s.s. exposed to the treated walls were highly resistant to deltamethrin (36% mortality rate). However, this population was susceptible to bendiocarb and pirimiphos methyl (100%) (Fig. 2). For each insecticide at least 80 specimens of *An. gambiae* were tested.

### Frequency of Leu–Phe *kdr* mutation

Molecular analysis of the wild *Anopheles* population from Dangbo showed very high frequency of *kdr* mutation (0.99) (49/50).

### Detoxification enzymes

**Fig. 3:**
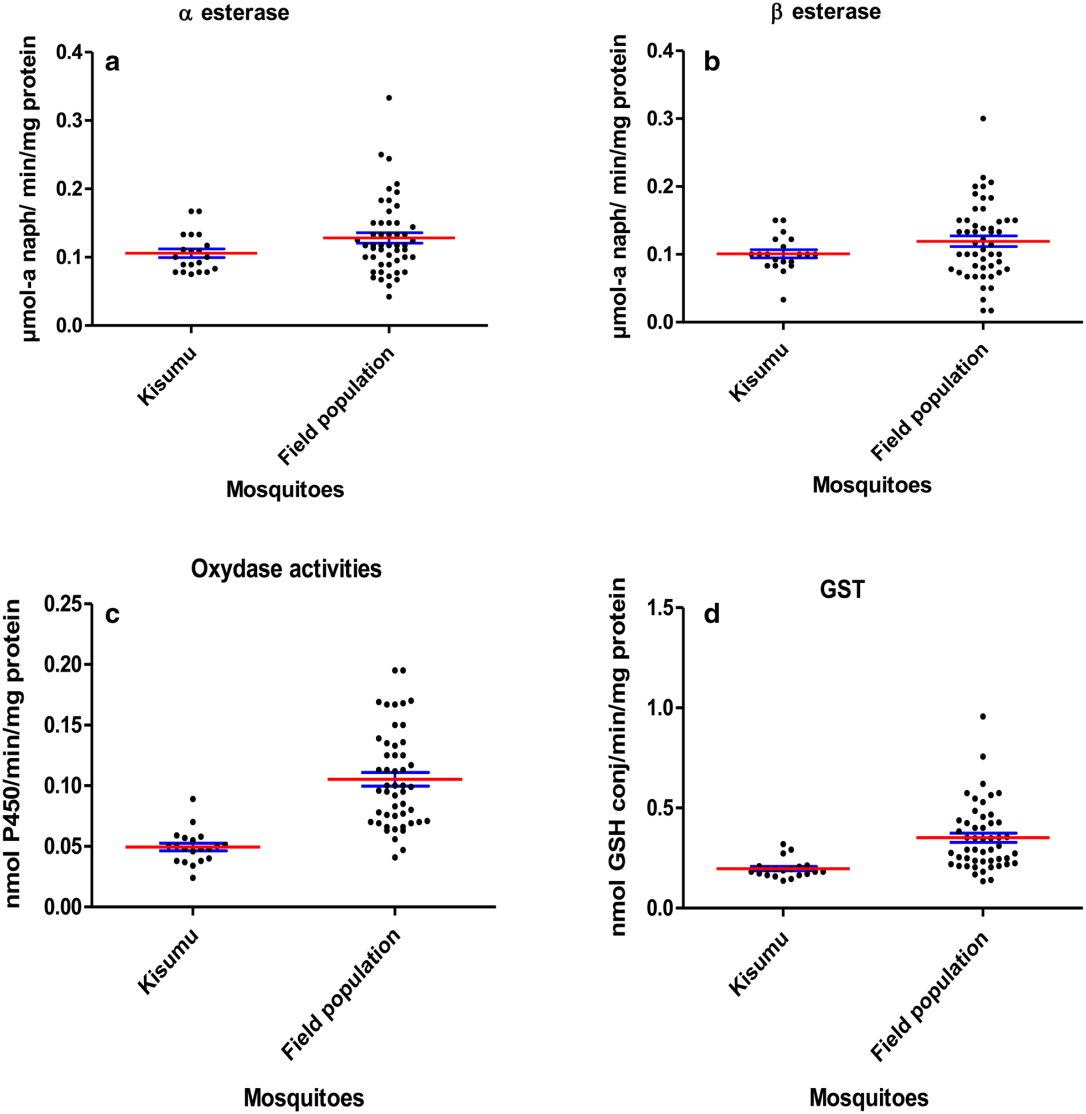
Level of detoxifying enzymes activities in *Anopheles gambiae* s.l. populations from Dangbo. **a** α esterase activity observed in wild mosquito populations. **b** β esterase activity observed in wild mosquito populations. **c** Oxydase activity observed in wild mosquito populations. **d** GST activity observed in wild mosquito population

Biochemical assays results showed that the activities of oxidases and GST were significantly higher in the wild *Anopheles* populations compared to Kisumu (p < 0.05) (Fig. 3c, d). No significant esterase activity was detected (p > 0.05) (Fig. 3a, b).

### Toxic effect and residual effect of treatments against laboratory susceptible “Kisumu” *Anopheles gambiae* population

**Fig. 4:**
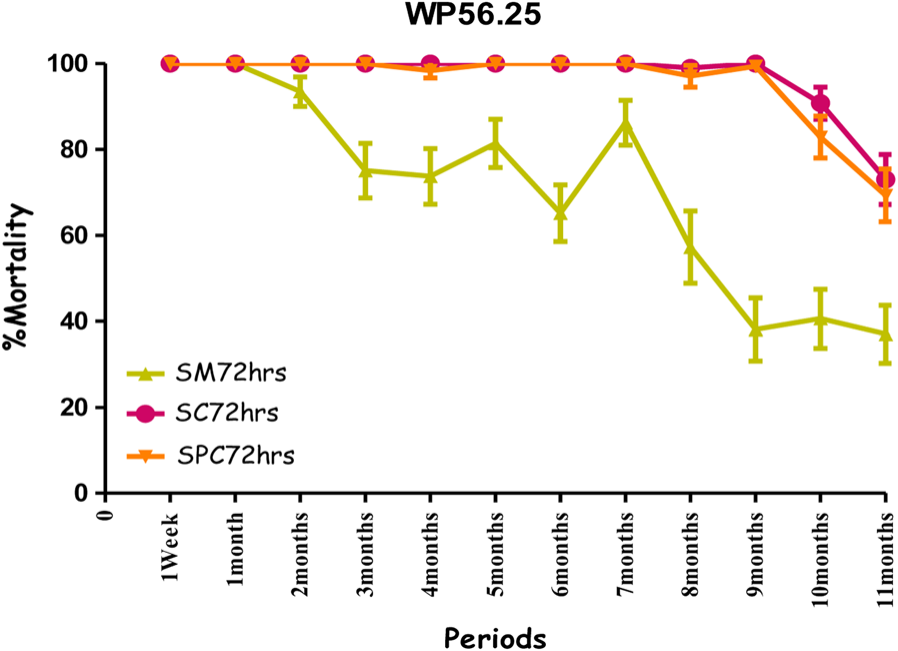
Efficacy represented by mortality rates per month, per observation time and per wall substrate of mixture clothianidin 200 mg/m^2^ + deltamethrin 25 mg/m^2^ (WP 56.25) against susceptible strain “Kisumu” *Anopheles gambiae* in operational conditions in Dangbo. *SM* smooth mud, *SC* smooth cement, *SPC* smooth paint cement

**Fig. 5:**
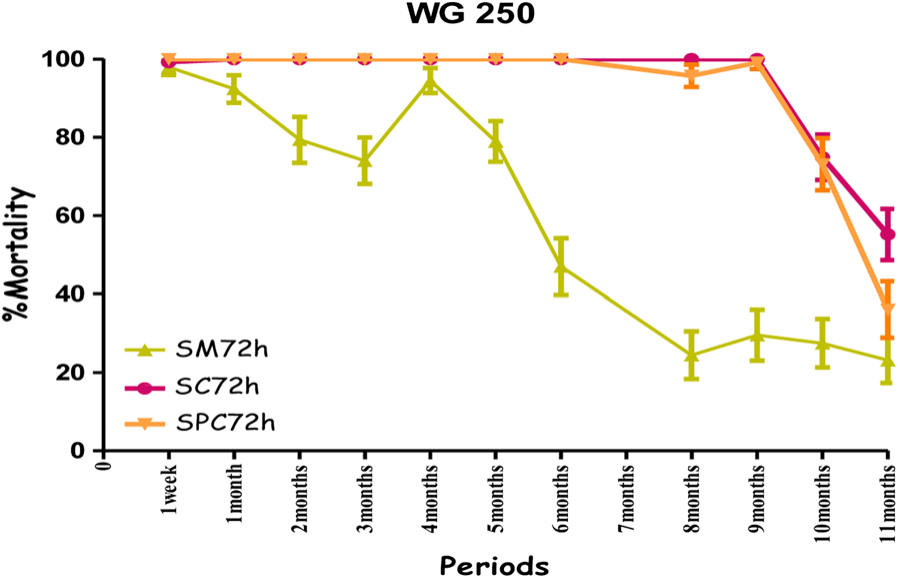
Efficacy represented by mortality rates per month, per observation time and per wall substrate of deltamethrin 25 mg/m^2^ (WG 250) against susceptible strain “Kisumu” *Anopheles gambiae* in operational conditions in Dangbo. *SM* smooth mud, *SC* smooth cement, *SPC* smooth paint cement

**Fig. 6:**
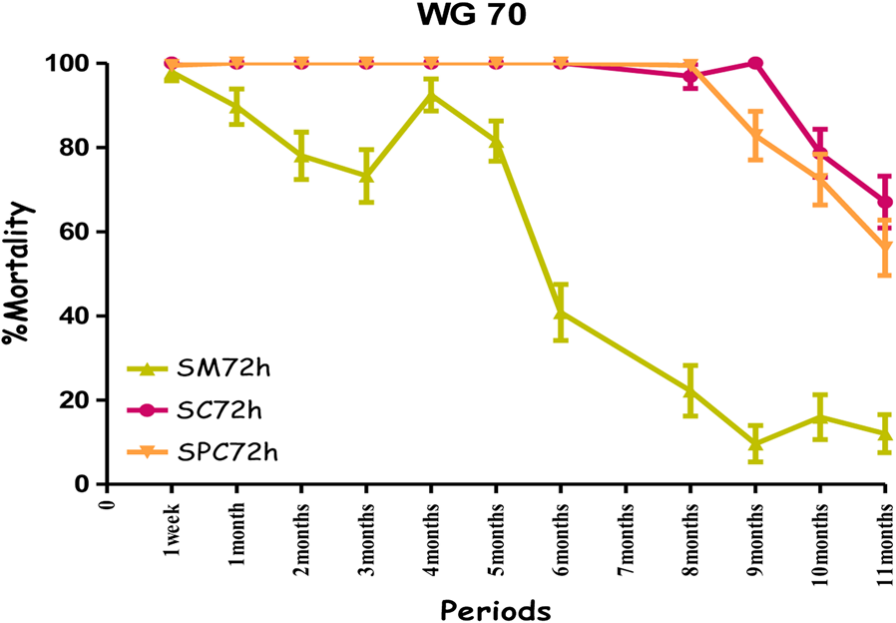
Efficacy represented by mortality rates per month, per observation time and per wall substrate of clothianidin 200 mg/m^2^ (WG 70) against susceptible strain “Kisumu” *Anopheles gambiae* in operational conditions in Dangbo. *SM* smooth mud, *SC* smooth cement, *SPC* smooth paint cement

All treatments showed the highest mortality rates on the cement walls (painted or non-painted). Mortality ranged from 80% to 100% for susceptible mosquitoes with no significant difference between the observation times (24 h, 48 h and 72 h) over a ten-month period with the combination Fludora^®^ Fusion and over a nine-month period for the individual insecticides deltamethrin WG 250 and clothianidin WG70 of the evaluation (Figs. 4, 5, 6 and Additional file 1). All treatments showed mortality rates under 80% WHO bio-efficacy threshold from month 3 to 11 on smooth mud walls (Figs. 4, 5 and 6).

Overall, the residual effect of Fludora^®^ Fusion with susceptible mosquitoes “Kisumu” was better than that of the individual insecticides deltamethrin WG 250 and clothianidin WG70 on smooth cement walls (painted or non-painted) and all treatments showed similar performance on smooth mud walls. The detailed results showing the killing effect with time of each insecticide formulation against Kisumu are provided (Additional file 1).

### Toxic effect and residual effect of treatments against wild insecticide resistant *Anopheles gambiae* s.s population

**Fig. 7:**
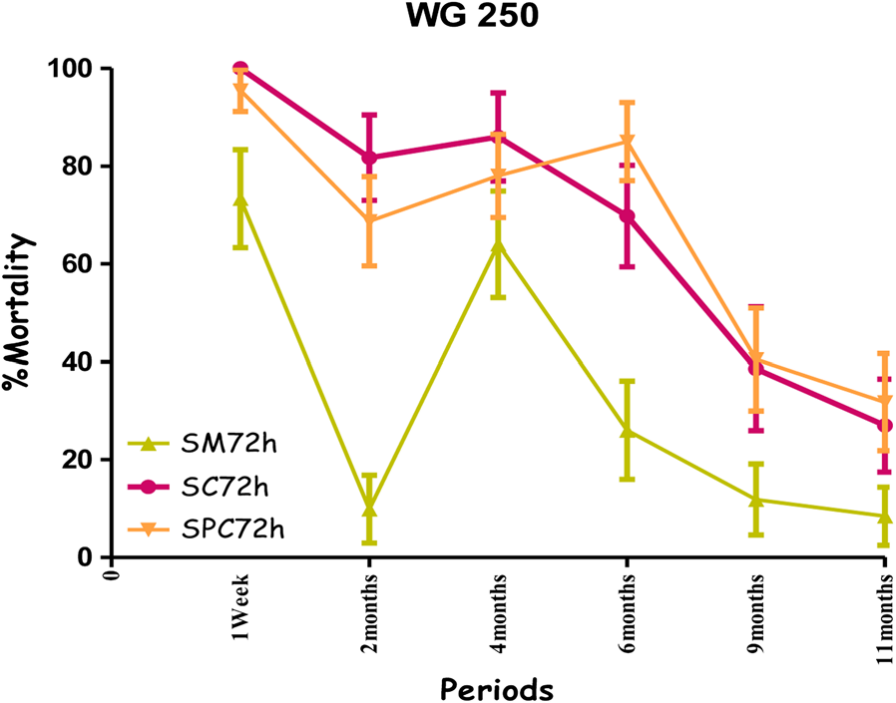
Efficacy represented by mortality rates per month, per observation time and per wall substrate of deltamethrin 25 mg/m^2^ (WG 250) against wild *Anopheles gambiae* s.s. in operational conditions in Dangbo. *SM* smooth mud, *SC* smooth cement, *SPC* smooth paint cement

The combination Fludora^®^ Fusion and clothianidin WG 70 on cement walls (both smooth and painted walls), showed high mortality rates exceeding the WHO threshold efficacy (80%) over a 9-month period against the wild population of *An. gambiae* from Dangbo, despite the high resistance of this population to pyrethroids. As was to be expected, the mortality rates observed with deltamethrin WG 250 were slightly lower (Fig. 7).

**Fig. 8:**
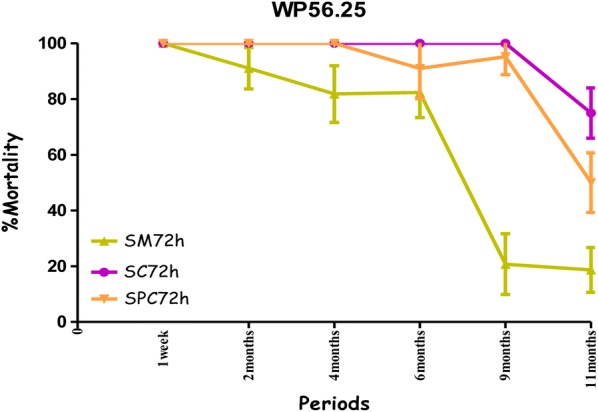
Efficacy represented by mortality rates per month, per observation time and per wall substrate of mixture clothianidin 200 mg/m^2^ + deltamethrin 25 mg/m^2^ (WP 56.25) against wild *Anopheles gambiae* s.s. in operational conditions in Dangbo. *SM* smooth mud, *SC* smooth cement, *SPC* smooth paint cement

**Fig. 9:**
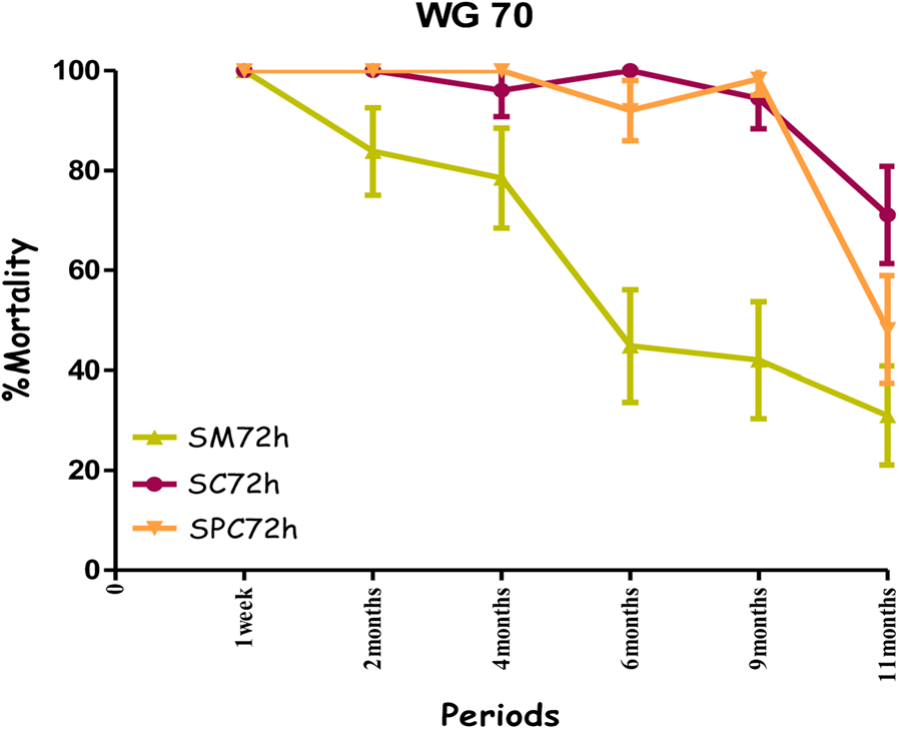
Efficacy represented by mortality rates per month, per observation time and per wall substrate of different formulations of clothianidin 200 mg/m^2^ (WG 70) against wild *Anopheles gambiae* s.s. in operational conditions in Dangbo. *SM* smooth mud, *SC* smooth cement, *SPC* smooth paint cement

The residual efficacy of Fludora^®^ Fusion and clothianidin WG70 on smooth mud walls was lower compared to the cement walls. When the mortality rates observed at 72 h post-exposure were taken into account the residual efficacy above the WHO threshold efficacy (80%) lasted for 6 months for the combination of Fludora^®^ Fusion and only 4 months for clothianidin WG70 (Figs. 8, 9). The mortality rates were under 80% for deltamethrin WG 250 during the whole period of the evaluation.

The mortality rates increased with time after exposure, with significant differences observed at 24 and 72 h—mostly in the smooth mud structures treated with Fludora^®^ Fusion and clothianidin WG 70 during the evaluation (Figs. 8, 9). The overall detailed results are summarized (Additional file 2).

## Discussion

The present study assessed in natural conditions but on a small scale, the efficacy and the residual effect of Fludora^®^ Fusion, a new neonicotinoid IRS formulation (mixture of clothianidin 200 mg ai/sqm + deltamethrin 25 mg ai/sqm) against populations of *Anopheles gambiae* s.s. susceptible and resistant to pyrethroids. Fludora^®^ Fusion showed good efficacy and the best residual effect of the other products tested (10 months on smooth cement wall and 6 months on smooth mud wall).

The results were interesting since deltamethrin 25 mg ai/sqm was ineffective irrespective of the kind of wall substrates against resistant Anopheles population from Dangbo during the whole study period. This observation reinforced the hypothesis that insecticide resistance constitutes a great threat to the success of malaria vector control methods. In Benin, pyrethroid resistance, carbamate resistance as well as the decrease of susceptibility to organophosphates have been reported in *An. gambiae* s.s. [4, 11–13].

Clothianidin is a novel neonicotinoid insecticide acting as an agonist at the nicotinic acetylcholine receptor (nAChR). Good efficacy of neonicotinoids against resistance Anopheles population has been demonstrated [14]. Few field studies have shown the efficacy of clothianidin in public health particularly for malaria vectors control. The results showed 10 months efficacy and residual effect of the insecticide combination Fludora^®^ Fusion on smooth cement walls—with some indications of improved efficacy compared to either of the individual components. The residual effect observed was higher (9 months) to that of insecticides that have been recommended by the WHO for indoor residual spraying [1]. On smooth mud walls, the residual effect of the Fludora^®^ Fusion combination was significantly lower, but still better than either of the individual components against an insecticide resistant strain. This reduction in bio-efficacy observed might be due to the porosity of the mud wall substrates. According to [15, 16], long-term efficacy was an issue of porosity of materials rather than the pH of materials. Also, many experimental huts studies conducted in Benin and at community level (indoor residual spray in Oueme and Atacora) have shown that the residual activity was very low with substrate of greater porosity [17, 18].

Overall, the Fludora^®^ Fusion combination showed some advantages over the individual components. Higher knock-down rates were observed with Fludora^®^ Fusion than clothianidin alone—which was to be expected since the presence of deltamethrin in the mixture contributed to the increase in knock-down effect. Pyrethroids are known to be insecticides with very good knock down properties. Also, the residual effect of the Fludora^®^ Fusion combination was slightly higher than that of clothianidin alone on both mud and cement substrates. This property will be of a great importance in several African rural areas where the majority of dwellings are made with mud. Existing recommended insecticides show short (almost 4 months) residual effect on mud walls [19]. In addition, the presence of deltamethrin in the mixture would certainly ameliorate the slow killing effect observed with clothianidin alone. The absence of cross resistance with recommended insecticide family (pyrethroids, organophosphates, organochlorines and carbamates) makes Fludora^®^ Fusion potential candidate for vector control, especially in areas where malaria vectors are highly resistant to insecticides. Other studies (WHOPES phase III) are needed to access the impact of IRS with Fludora^®^ Fusion on malaria transmission indicators.

## Conclusion

Fludora^®^ Fusion and clothianidin showed very good performances against *An. gambiae* s.s. populations, which are highly resistant to pyrethroids. However, the Fludora^®^ Fusion combination seems to last on cement and mud substrates longer than clothianidin alone. It will be interesting to perform a normal operational indoor residual spraying i.e. WHOPES phase III in areas where Anopheles are resistant to carbamates or organophosphates to compare their efficacies and residual effects and to assess the impact of these promising compounds on malaria transmission indicators.

## Additional files

**Additional file 1: Table S1.** Detailed data of the efficacy represented by the mortality rates and the Knock down rates 30 min after exposition per time and per wall substrate of mixture clothianidin 200 mg/m² + deltamethrin 25 mg/m², clothianidin 200 mg/m² and deltamethrin 25 mg/m² against laboratory susceptible strain “kisumu” in operational conditions in Dangbo.

**Additional file 2: Table S2.** Efficacy represented by mortality rates and Knock down 30 min after exposition per time and per wall substrate of mixture clothianidin 200 mg/m² + deltamethrin 25 mg/m², clothianidin 200 mg/m² and deltamethrin 25 mg/m² against wild *An. gambiae* s.s. in operational conditions in Dangbo.
