# Supplementary material for: Small-scale field evaluation of the efficacy and residual effect of Fludora® Fusion (mixture of clothianidin and deltamethrin) against susceptible and resistant Anopheles gambiae populations from Benin, West Africa
Source: Malar J. 2018 Dec 29;17:484. doi: 10.1186/s12936-018-2633-6 (PMC6311023; doi:10.1186/s12936-018-2633-6)
Supplement: Supplementary file 1 — Additional file 1: Table S1. Detailed data of the efficacy represented by the mortality rates and the Knock down rates 30 min after exposition per time and per wall substrate of mixture clothianidin 200 mg/m² + deltamethrin 25 mg/m², clothianidin 200 mg/m² and deltamethrin 25 mg/m² against laboratory susceptible strain “kisumu” in operational conditions in Dangbo. [file 12936_2018_2633_MOESM1_ESM.doc]

**Table S1 : Detailed data of the efficacy represented by the mortality rates and the Knock down rates 30 min after exposition per time and per wall substrate of mixture Clothianidin 200 mg/m² + Deltamethrin 25 mg/m², Clothianidin 200 mg/m² and Deltamethrin 25 mg/m² against laboratory susceptible strain “kisumu” in operational conditions in Dangbo**

| **Treatment** | **Structure** | **Month** | **KD 30 min** | **Mort 24h** | **Mort 48h** | **Mort 72h** | **Total tested** | **%KD 30 min** | **%Mort 24h** | **%Mort 48h** | **%Mort 72h** |
| --- | --- | --- | --- | --- | --- | --- | --- | --- | --- | --- | --- |
| **Control** | **SM** | **1W** | *0* | *1* | *1* | *1* | *90* | *0* | *1,11* | *1,11* | *1* |
|  | **SM** | **1** | *0* | *1* | *1* | *1* | *80* | *0* | *1,25* | *1,25* | *1,25* |
|  | **SM** | **2** | *0* | *2* | *2* | *2* | *73* | *0* | *2,74* | *2,74* | *2,74* |
|  | **SM** | **3** | *0* | *1* | *5* | *7* | *81* | *0* | *1* | *6,17* | *8,64* |
|  | **SM** | **4** | *0* | *1* | *5* | *7* | *89* | *0* | *1,12* | *5,62* | *7,87* |
|  | **SM** | **5** | *0* | *0* | *0* | *2* | *84* | *0* | *0* | *0* | *2,38* |
|  | **SM** | **6** | *0* | *0* | *4* | *5* | *80* | *0* | *0* | *5* | *6,25* |
|  | **SM** | **7** | *0* | *5* | *7* | *7* | *77* | *0* | *6,49* | *9,09* | *9,09* |
|  | **SM** | **8** | *0* | *9* | *10* | *10* | *76* | *0* | *11,84* | *13,16* | *13,16* |
|  | **SM** | **9** | *0* | *5* | *7* | *7* | *63* | *0* | *7,94* | *11,11* | *11,11* |
|  | **SM** | **10** | *0* | *2* | *2* | *2* | *72* | *0* | *2,78* | *2,78* | *2,78* |
|  | **SM** | **11** | *0* | *2* | *2* | *2* | *84* | *0* | *2,38* | *2,38* | *2,38* |
| **Control** | **SC** | **1W** | *0* | *1* | *1* | *1* | *77* | *0* | *1,30* | *1,30* | *1,30* |
|  | **SC** | **1** | *0* | *0* | *0* | *0* | *80* | *0* | *0* | *0* | *0* |
|  | **SC** | **2** | *1* | *1* | *1* | *1* | *77* | *1,30* | *1,30* | *1,30* | *1,30* |
|  | **SC** | **3** | *0* | *3* | *8* | *8* | *95* | *0* | *3,16* | *8,42* | *8,42* |
|  | **SC** | **4** | *1* | *1* | *1* | *1* | *81* | *1,23* | *1,23* | *1,23* | *1,23* |
|  | **SC** | **5** | *0* | *0* | *1* | *1* | *85* | *0* | *0* | *1,18* | *1,18* |
|  | **SC** | **6** | *0* | *0* | *2* | *4* | *95* | *0* | *0* | *2,11* | *4,21* |
|  | **SC** | **7** | *0* | *2* | *2* | *3* | *83* | *0* | *2,41* | *2,41* | *3,61* |
|  | **SC** | **8** | *0* | *7* | *7* | *9* | *84* | *0* | *8,33* | *8,33* | *10,71* |
|  | **SC** | **9** | *0* | *1* | *3* | *3* | *64* | *0* | *1,56* | *4,69* | *4,69* |
|  | **SC** | **10** | *0* | *1* | *2* | *2* | *77* | *0* | *1,30* | *2,60* | *2,60* |
|  | **SC** | **11** | *0* | *0* | *0* | *0* | *82* | *0* | *0* | *0* | *0* |
| **Control** | **SPC** | **1W** | *0* | *4* | *4* | *4* | *91* | *0* | *4,40* | *4,40* | *4,40* |
|  | **SPC** | **1** | *0* | *1* | *1* | *1* | *71* | *0* | *1,41* | *1,41* | *1,41* |
|  | **SPC** | **2** | *0* | *2* | *2* | *2* | *69* | *0* | *2,90* | *2,90* | *2,90* |
|  | **SPC** | **3** | *0* | *0* | *7* | *10* | *81* | *0* | *0* | *8,64* | *12,35* |
|  | **SPC** | **4** | *0* | *0* | *7* | *10* | *89* | *0* | *0* | *7,87* | *11,24* |
|  | **SPC** | **5** | *0* | *0* | *0* | *2* | *76* | *0* | *0* | *0* | *2,63* |
|  | **SPC** | **6** | *0* | *2* | *4* | *4* | *92* | *0* | *2,17* | *4,35* | *4,35* |
|  | **SPC** | **7** | *0* | *1* | *2* | *4* | *70* | *0* | *1,43* | *2,86* | *5,71* |
|  | **SPC** | **8** | *0* | *10* | *10* | *11* | *71* | *0* | *14,08* | *14,08* | *15,49* |
|  | **SPC** | **9** | *0* | *3* | *5* | *5* | *70* | *0* | *4,29* | *7,14* | *7,14* |
|  | **SPC** | **10** | *0* | *1* | *2* | *2* | *85* | *0* | *1* | *2,35* | *2,35* |
|  | **SPC** | **11** | *0* | *1* | *2* | *2* | *76* | *0* | *1* | *2,63* | *2,63* |
| **WP 56.25** | **SM** | **1W** | *178* | *182* | *184* | *184* | *184* | *96,74* | *98,91* | *100* | *100* |
|  | **SM** | **1** | *145* | *173* | *180* | *183* | *183* | *79,23* | *94,54* | *98,36* | *100* |
|  | **SM** | **2** | *132* | *165* | *182* | *187* | *200* | *66* | *82,5* | *91* | *93,5* |
|  | **SM** | **3** | *66* | *98* | *121* | *139* | *180* | *36,67* | *54,44* | *65,07* | *75,07* |
|  | **SM** | **4** | *86* | *93* | *111* | *132* | *179* | *48,04* | *51,96* | *62,01* | *73,74* |
|  | **SM** | **5** | *32* | *102* | *135* | *149* | *183* | *17,49* | *55,74* | *73,77* | *81,42* |
|  | **SM** | **6** | *125* | *114* | *127* | *134* | *199* | *62,81* | *57,29* | *63,82* | *65,16* |
|  | **SM** | **7** | *59* | *90* | *127* | *147* | *168* | *35,12* | *50,35* | *73,15* | *86,25* |
|  | **SM** | **8** | *43* | *47* | *55* | *83* | *132* | *32,58* | *26,96* | *32,83* | *57,25* |
|  | **SM** | **9** | *31* | *65* | *74* | *76* | *169* | *18,34* | *33,16* | *36,76* | *38,09* |
|  | **SM** | **10** | *25* | *62* | *72* | *80* | *197* | *12,69* | *31* | *36,55* | *40,61* |
|  | **SM** | **11** | *25* | *53* | *63* | *73* | *197* | *12,69* | *27* | *31,98* | *37* |
|  | **SC** | **1W** | *137* | *238* | *238* | *238* | *238* | *57,56* | *100* | *100* | *100* |
|  | **SC** | **1** | *172* | *199* | *199* | *199* | *199* | *86,43* | *100* | *100* | *100* |
|  | **SC** | **2** | *135* | *177* | *177* | *177* | *177* | *76,27* | *100* | *100* | *100* |
|  | **SC** | **3** | *132* | *166* | *166* | *166* | *166* | *79,52* | *100* | *100* | *100* |
|  | **SC** | **4** | *211* | *222* | *226* | *226* | *226* | *93,36* | *98,23* | *100* | *100* |
|  | **SC** | **5** | *203* | *203* | *203* | *203* | *203* | *100* | *100* | *100* | *100* |
|  | **SC** | **6** | *206* | *218* | *218* | *218* | *218* | *94,50* | *100* | *100* | *100* |
|  | **SC** | **7** | *138* | *168* | *170* | *170* | *170* | *81,18* | *98,82* | *100* | *100* |
|  | **SC** | **8** | *145* | *215* | *226* | *226* | *228* | *63,60* | *93,78* | *99,04* | *99,02* |
|  | **SC** | **9** | *180* | *241* | *241* | *241* | *241* | *74,69* | *100* | *100* | *100* |
|  | **SC** | **10** | *129* | *161* | *189* | *207* | *228* | *56,58* | *70,61* | *83* | *90,79* |
|  | **SC** | **11** | *101* | *133* | *149* | *164* | *225* | *44,89* | *59,11* | *66* | *73* |
|  | **SPC** | **1W** | *166* | *222* | *223* | *223* | *223* | *74,44* | *99,55* | *100* | *100* |
|  | **SPC** | **1** | *188* | *214* | *214* | *214* | *214* | *87,85* | *100* | *100* | *100* |
|  | **SPC** | **2** | *175* | *197* | *197* | *197* | *197* | *88,83* | *100* | *100* | *100* |
|  | **SPC** | **3** | *202* | *213* | *215* | *216* | *216* | *93,52* | *98,61* | *99,49* | *100* |
|  | **SPC** | **4** | *184* | *204* | *206* | *206* | *209* | *88,04* | *97,61* | *98,44* | *98,38* |
|  | **SPC** | **5** | *196* | *205* | *205* | *205* | *205* | *95,61* | *100* | *100* | *100* |
|  | **SPC** | **6** | *157* | *164* | *164* | *164* | *164* | *95,73* | *100* | *100* | *100* |
|  | **SPC** | **7** | *167* | *177* | *177* | *177* | *177* | *94,35* | *100* | *100* | *100* |
|  | **SPC** | **8** | *82* | *153* | *158* | *160* | *164* | *50* | *92,19* | *95,74* | *97,11* |
|  | **SPC** | **9** | *134* | *178* | *182* | *182* | *183* | *73,22* | *97,27* | *99,41* | *99,41* |
|  | **SPC** | **10** | *83* | *133* | *158* | *189* | *228* | *36,40* | *58,33* | *69,30* | *82,89* |
|  | **SPC** | **11** | *53* | *95* | *121* | *149* | *215* | *24,65* | *44,19* | *56,28* | *69,30* |
| **WG 250** | **SM** | **1W** | *147* | *185* | *187* | *189* | *193* | *76,17* | *95,85* | *96,89* | *97,93* |
|  | **SM** | **1** | *112* | *185* | *191* | *205* | *222* | *50,45* | *83,33* | *86,04* | *92,34* |
|  | **SM** | **2** | *89* | *127* | *140* | *146* | *184* | *48,37* | *69,02* | *76,09* | *79,35* |
|  | **SM** | **3** | *87* | *149* | *145* | *161* | *211* | *41,23* | *70,62* | *66,66* | *74,00* |
|  | **SM** | **4** | *53* | *157* | *174* | *186* | *196* | *27,04* | *80,10* | *88,11* | *94,46* |
|  | **SM** | **5** | *88* | *171* | *183* | *187* | *237* | *37,13* | *72,15* | *77,22* | *78,90* |
|  | **SM** | **6** | *43* | *67* | *82* | *92* | *183* | *23,50* | *36,61* | *44,81* | *46,96* |
|  | **SM** | **7** | *60* | *166* | *209* | *219* | *243* | *24,69* | *66,11* | *84,61* | *89,14* |
|  | **SM** | **8** | *34* | *61* | *63* | *66* | *192* | *17,71* | *22,61* | *22,63* | *24,43* |
|  | **SM** | **9** | *6* | *46* | *58* | *71* | *190* | *3,16* | *17,68* | *21,84* | *29,54* |
|  | **SM** | **10** | *26* | *39* | *55* | *54* | *200* | *13* | *19,50* | *27,5* | *27* |
|  | **SM** | **11** | *24* | *29* | *43* | *47* | *203* | *12* | *14,29* | *21* | *23,15* |
|  | **SC** | **1W** | *251* | *252* | *252* | *252* | *254* | *98,82* | *99,21* | *99,21* | *99,21* |
|  | **SC** | **1** | *205* | *208* | *208* | *208* | *208* | *98,56* | *100* | *100* | *100* |
|  | **SC** | **2** | *211* | *220* | *220* | *220* | *220* | *95,91* | *100* | *100* | *100* |
|  | **SC** | **3** | *172* | *182* | *182* | *182* | *182* | *94,51* | *100* | *100* | *100* |
|  | **SC** | **4** | *178* | *192* | *192* | *192* | *192* | *92,71* | *100* | *100* | *100* |
|  | **SC** | **5** | *199* | *221* | *221* | *221* | *221* | *90,05* | *100* | *100* | *100* |
|  | **SC** | **6** | *134* | *213* | *216* | *216* | *216* | *62,04* | *98,61* | *100* | *100* |
|  | **SC** | **7** | *163* | *206* | *210* | *213* | *213* | *76,53* | *96,71* | *98,59* | *100* |
|  | **SC** | **8** | *119* | *192* | *193* | *196* | *196* | *60,71* | *97,77* | *98,33* | *100* |
|  | **SC** | **9** | *156* | *171* | *171* | *171* | *171* | *91,23* | *100* | *100* | *100* |
|  | **SC** | **10** | *100* | *142* | *150* | *161* | *215* | *46,51* | *66,05* | *69,77* | *74,88* |
|  | **SC** | **11** | *96* | *116* | *119* | *123* | *223* | *43,05* | *52,02* | *53,36* | *55,16* |
|  | **SPC** | **1W** | *229* | *229* | *229* | *229* | *229* | *100* | *100* | *100* | *100* |
|  | **SPC** | **1** | *128* | *128* | *128* | *128* | *128* | *100* | *100* | *100* | *100* |
|  | **SPC** | **2** | *233* | *234* | *234* | *234* | *234* | *99,57* | *100* | *100* | *100* |
|  | **SPC** | **3** | *227* | *228* | *228* | *228* | *228* | *99,56* | *100* | *100* | *100* |
|  | **SPC** | **4** | *203* | *206* | *206* | *206* | *206* | *98,54* | *100* | *100* | *100* |
|  | **SPC** | **5** | *212* | *214* | *214* | *214* | *214* | *99,07* | *100* | *100* | *100* |
|  | **SPC** | **6** | *187* | *202* | *204* | *204* | *204* | *91,67* | *99,02* | *100* | *100* |
|  | **SPC** | **7** | *197* | *201* | *201* | *201* | *201* | *98,01* | *100* | *100* | *100* |
|  | **SPC** | **8** | *114* | *178* | *186* | *186* | *193* | *59,07* | *90,95* | *95,78* | *95,71* |
|  | **SPC** | **9** | *93* | *119* | *119* | *120* | *121* | *76,86* | *98,35* | *98,22* | *99,11* |
|  | **SPC** | **10** | *79* | *108* | *121* | *125* | *171* | *46,20* | *63,16* | *70,76* | *73,10* |
|  | **SPC** | **11** | *59* | *43* | *56* | *62* | *172* | *34,30* | *25* | *32,56* | *36,05* |
| **WG 70** | **SM** | **1W** | *11* | *159* | *180* | *185* | *189* | *5,82* | *84,13* | *95,24* | *97,88* |
|  | **SM** | **1** | *5* | *111* | *156* | *182* | *203* | *2,46* | *54,68* | *76,85* | *89,66* |
|  | **SM** | **2** | *17* | *92* | *111* | *163* | *209* | *8,13* | *44,02* | *53,11* | *77,99* |
|  | **SM** | **3** | *5* | *63* | *80* | *145* | *192* | *2,60* | *32,81* | *37,83* | *73,21* |
|  | **SM** | **4** | *19* | *138* | *153* | *174* | *187* | *10,16* | *73,80* | *80,74* | *92,45* |
|  | **SM** | **5** | *70* | *167* | *178* | *207* | *254* | *27,56* | *65,75* | *70,08* | *81,50* |
|  | **SM** | **6** | *3* | *57* | *80* | *93* | *209* | *1,44* | *27,27* | *38,28* | *40,80* |
|  | **SM** | **7** | *11* | *201* | *231* | *242* | *257* | *4,28* | *76,70* | *88,87* | *93,58* |
|  | **SM** | **8** | *3* | *44* | *49* | *60* | *185* | *1,62* | *13,55* | *15,35* | *22,19* |
|  | **SM** | **9** | *2* | *17* | *29* | *35* | *178* | *1,12* | *1,75* | *5,83* | *9,62* |
|  | **SM** | **10** | *10* | *18* | *25* | *29* | *182* | *5,49* | *9,89* | *13,74* | *15,93* |
|  | **SM** | **11** | *17* | *14* | *20* | *24* | *200* | *8,50* | *7* | *10* | *12* |
|  | **SC** | **1W** | *135* | *210* | *212* | *215* | *215* | *62,79* | *97,67* | *98,60* | *100* |
|  | **SC** | **1** | *170* | *170* | *170* | *170* | *170* | *100* | *100* | *100* | *100* |
|  | **SC** | **2** | *172* | *195* | *195* | *195* | *195* | *88,21* | *100* | *100* | *100* |
|  | **SC** | **3** | *162* | *194* | *194* | *194* | *194* | *83,51* | *100* | *100* | *100* |
|  | **SC** | **4** | *152* | *168* | *168* | *168* | *168* | *90,48* | *100* | *100* | *100* |
|  | **SC** | **5** | *92* | *151* | *157* | *157* | *157* | *58,60* | *96,18* | *100* | *100* |
|  | **SC** | **6** | *124* | *157* | *157* | *157* | *157* | *78,98* | *100* | *100* | *100* |
|  | **SC** | **7** | *67* | *160* | *163* | *164* | *165* | *40,61* | *96,97* | *98,79* | *99,39* |
|  | **SC** | **8** | *72* | *135* | *137* | *139* | *143* | *50,35* | *93,90* | *95,42* | *96,87* |
|  | **SC** | **9** | *103* | *130* | *130* | *130* | *130* | *79,23* | *100* | *100* | *100* |
|  | **SC** | **10** | *70* | *126* | *144* | *158* | *201* | *34,83* | *62,69* | *71,64* | *78,61* |
|  | **SC** | **11** | *39* | *95* | *112* | *149* | *223* | *17,49* | *42,60* | *50,22* | *67* |
|  | **SPC** | **1W** | *9* | *181* | *182* | *182* | *183* | *4,92* | *98,91* | *99,45* | *99,45* |
|  | **SPC** | **1** | *34* | *103* | *103* | *103* | *103* | *33,01* | *100* | *100* | *100* |
|  | **SPC** | **2** | *8* | *176* | *176* | *176* | *176* | *4,55* | *100* | *100* | *100* |
|  | **SPC** | **3** | *13* | *103* | *108* | *109* | *109* | *11,93* | *94,50* | *99* | *100* |
|  | **SPC** | **4** | *12* | *107* | *108* | *109* | *109* | *11,01* | *98,17* | *99* | *100* |
|  | **SPC** | **5** | *17* | *147* | *148* | *148* | *148* | *11,49* | *99,32* | *100* | *100* |
|  | **SPC** | **6** | *31* | *163* | *163* | *163* | *163* | *19,02* | *100* | *100* | *100* |
|  | **SPC** | **7** | *31* | *198* | *199* | *199* | *199* | *15,58* | *99,50* | *100* | *100* |
|  | **SPC** | **8** | *24* | *178* | *203* | *212* | *213* | *11,27* | *80,87* | *94,54* | *99,44* |
|  | **SPC** | **9** | *16* | *98* | *125* | *137* | *163* | *9,82* | *60,12* | *74,89* | *82,82* |
|  | **SPC** | **10** | *21* | *97* | *125* | *152* | *210* | *10* | *46,19* | *59,52* | *72,38* |
|  | **SPC** | **11** | *25* | *74* | *102* | *123* | *219* | *11* | *33,79* | *46,58* | *56,16* |

1W = 1 week after ; SM = smooth mud, SC = smooth cement ; SPC = smooth painted cement ; WP 56.25 : mixture Clothianidin 200 mg m.a/m² + Deltamethrin 25 mg m.a/m² ; WG 250 : Deltamethrin 25 mg m.a/m² et WG70 : Clothianidin 200 mg m.a/m².
