# Supplementary material for: Small-scale field evaluation of the efficacy and residual effect of Fludora® Fusion (mixture of clothianidin and deltamethrin) against susceptible and resistant Anopheles gambiae populations from Benin, West Africa
Source: Malar J. 2018 Dec 29;17:484. doi: 10.1186/s12936-018-2633-6 (PMC6311023; doi:10.1186/s12936-018-2633-6)
Supplement: Supplementary file 2 — Additional file 2: Table S2. Efficacy represented by mortality rates and Knock down 30 min after exposition per time and per wall substrate of mixture clothianidin 200 mg/m² + deltamethrin 25 mg/m², clothianidin 200 mg/m² and deltamethrin 25 mg/m² against wild An. gambiae s.s. in operational conditions in Dangbo. [file 12936_2018_2633_MOESM2_ESM.doc]

**Table S2 :** Efficacy represented by mortality rates and Knock down 30 min after exposition per time and per wall substrate of mixture Clothianidin 200 mg/m² + Deltamethrin 25 mg/m², Clothianidin 200 mg/m² and Deltamethrin 25 mg/m² against wild *An. gambiae* s.s. in operational conditions in Dangbo

| **Treatment** | **Structure** | **Month** | **KD 30 min** | **Mort 24h** | **Mort 48h** | **Mort 72h** | **Total tested** | **%KD 30 min** | **%Mort 24h** | **%Mort 48h** | **%Mort 72h** |
| --- | --- | --- | --- | --- | --- | --- | --- | --- | --- | --- | --- |
| **Control** | **SM** | **1W** | *0* | *0* | *0* | *0* | *33* | *0* | *0* | *0* | *0* |
|  | **SM** | **2** | *0* | *0* | *0* | *1* | *42* | *0* | *0* | *0* | *2,38* |
|  | **SM** | **4** | *0* | *0* | *0* | *0* | *43* | *0* | *0* | *0* | *0* |
|  | **SM** | **6** | *0* | *0* | *3* | *3* | *37* | *0* | *0* | *8,11* | *8,11* |
|  | **SM** | **9** | *0* | *1* | *1* | *1* | *82* | *0* | *1,22* | *1,22* | *1,22* |
|  | **SM** | **11** | *1* | *1* | *1* | *2* | *85* | *1* | *1,18* | *1,18* | *2,35* |
|  | **SC** | **1W** | *0* | *0* | *0* | *0* | *40* | *0* | *0* | *0* | *0* |
|  | **SC** | **2** | *0* | *1* | *1* | *2* | *39* | *0* | *2,56* | *2,56* | *5,13* |
|  | **SC** | **4** | *0* | *1* | *1* | *1* | *31* | *0* | *3,23* | *3,23* | *3,23* |
|  | **SC** | **6** | *0* | *2* | *3* | *4* | *34* | *0* | *5,88* | *8,82* | *11,76* |
|  | **SC** | **9** | *0* | *1* | *1* | *1* | *77* | *0* | *1,30* | *1,30* | *1,30* |
|  | **SC** | **11** | *0* | *1* | *1* | *1* | *89* | *0* | *1,12* | *1,12* | *1,12* |
|  | **SPC** | **1W** | *0* | *4* | *4* | *4* | *41* | *0* | *9,76* | *9,76* | *9,76* |
|  | **SPC** | **2** | *0* | *0* | *1* | *1* | *48* | *0* | *0* | *2,08* | *2,08* |
|  | **SPC** | **4** | *0* | *0* | *0* | *0* | *35* | *0* | *0* | *0* | *0* |
|  | **SPC** | **6** | *0* | *2* | *2* | *5* | *43* | *0* | *5* | *5* | *11,63* |
|  | **SPC** | **9** | *0* | *1* | *1* | *2* | *80* | *0* | *1,25* | *1,25* | *2,50* |
|  | **SPC** | **11** | *0* | *1* | *1* | *1* | *80* | *0* | *1,25* | *1,25* | *1,25* |
| **WP 56.25** | **SM** | **1W** | *5* | *80* | *80* | *80* | *80* | *6,25* | *100* | *100* | *100* |
|  | **SM** | **2** | *0* | *44* | *49* | *51* | *56* | *0* | *79* | *87,50* | *91,07* |
|  | **SM** | **4** | *1* | *36* | *42* | *45* | *55* | *1,82* | *65,45* | *76,36* | *81,82* |
|  | **SM** | **6** | *0* | *27* | *50* | *57* | *68* | *0* | *39,71* | *71,19* | *82,40* |
|  | **SM** | **9** | *0* | *4* | *10* | *11* | *53* | *0* | *7,55* | *18,87* | *20,75* |
|  | **SM** | **11** | *3* | *3* | *6* | *17* | *91* | *3* | *3,30* | *6,59* | *18,68* |
|  | **SC** | **1W** | *58* | *89* | *89* | *89* | *89* | *65,17* | *100* | *100* | *100* |
|  | **SC** | **2** | *16* | *65* | *65* | *65* | *65* | *24,62* | *100* | *100* | *100* |
|  | **SC** | **4** | *22* | *66* | *66* | *66* | *66* | *33* | *100* | *100* | *100* |
|  | **SC** | **6** | *14* | *64* | *66* | *66* | *66* | *21,21* | *96,97* | *100* | *100* |
|  | **SC** | **9** | *23* | *80* | *80* | *80* | *80* | *29* | *100* | *100* | *100* |
|  | **SC** | **11** | *14* | *45* | *55* | *66* | *88* | *16* | *51* | *63* | *75* |
|  | **SPC** | **1W** | *51* | *89* | *89* | *89* | *89* | *57,30* | *100* | *100* | *100* |
|  | **SPC** | **2** | *3* | *62* | *62* | *62* | *62* | *4,84* | *100* | *100* | *100* |
|  | **SPC** | **4** | *23* | *84* | *86* | *86* | *86* | *27* | *98* | *100* | *100* |
|  | **SPC** | **6** | *1* | *22* | *24* | *24* | *26* | *4* | *85* | *92* | *91* |
|  | **SPC** | **9** | *9* | *40* | *40* | *40* | *42* | *21,43* | *95,24* | *95,24* | *95,24* |
|  | **SPC** | **11** | *18* | *32* | *37* | *42* | *84* | *21* | *38,10* | *44,05* | *50* |
| **WG 250** | **SM** | **1W** | *17* | *19* | *38* | *55* | *75* | *22,67* | *25,33* | *50,67* | *73,33* |
|  | **SM** | **2** | *1* | *7* | *7* | *7* | *71* | *1,41* | *10* | *9,86* | *9,86* |
|  | **SM** | **4** | *5* | *40* | *46* | *48* | *75* | *6,67* | *53,33* | *61,33* | *64* |
|  | **SM** | **6** | *6* | *15* | *18* | *19* | *73* | *8,22* | *20,55* | *24,66* | *26,03* |
|  | **SM** | **9** | *0* | *4* | *6* | *9* | *76* | *0* | *5,26* | *7,89* | *11,84* |
|  | **SM** | **11** | *5* | *4* | *6* | *7* | *83* | *6* | *5* | *7,23* | *8,43* |
|  | **SC** | **1W** | *45* | *76* | *78* | *84* | *84* | *53,57* | *90,48* | *92,86* | *100* |
|  | **SC** | **2** | *51* | *55* | *58* | *62* | *75* | *68* | *73,33* | *77,33* | *81,73* |
|  | **SC** | **4** | *10* | *40* | *46* | *49* | *57* | *17,54* | *70,18* | *80,70* | *85,96* |
|  | **SC** | **6** | *26* | *47* | *53* | *55* | *75* | *35* | *63* | *67,83* | *69,78* |
|  | **SC** | **9** | *3* | *14* | *21* | *22* | *57* | *5,26* | *25* | *36,84* | *38,60* |
|  | **SC** | **11** | *6* | *13* | *21* | *23* | *84* | *7* | *15* | *25* | *27* |
|  | **SPC** | **1W** | *32* | *61* | *74* | *85* | *89* | *35,96* | *68,54* | *83,15* | *95,51* |
|  | **SPC** | **2** | *69* | *60* | *62* | *68* | *99* | *69,70* | *61* | *62,63* | *68,69* |
|  | **SPC** | **4** | *30* | *60* | *66* | *71* | *91* | *32,97* | *66* | *73* | *78,02* |
|  | **SPC** | **6** | *43* | *53* | *61* | *66* | *76* | *56,58* | *70* | *80,26* | *85* |
|  | **SPC** | **9** | *0* | *18* | *29* | *34* | *84* | *0* | *21,43* | *34,52* | *40,48* |
|  | **SPC** | **11** | *1* | *14* | *24* | *27* | *85* | *1* | *16* | *28,24* | *31,76* |
| **WG 70** | **SM** | **1W** | *2* | *54* | *61* | *67* | *67* | *2,99* | *80,60* | *91,04* | *100* |
|  | **SM** | **2** | *0* | *51* | *54* | *57* | *68* | *0* | *75* | *79,41* | *83,82* |
|  | **SM** | **4** | *0* | *40* | *48* | *51* | *65* | *0* | *61,54* | *73,85* | *78,46* |
|  | **SM** | **6** | *0* | *15* | *33* | *37* | *75* | *0* | *20* | *39,06* | *44,86* |
|  | **SM** | **9** | *0* | *14* | *26* | *29* | *69* | *0* | *20,29* | *37,68* | *42,03* |
|  | **SM** | **11** | *3* | *14* | *22* | *26* | *84* | *3,57* | *17* | *26,19* | *30,95* |
|  | **SC** | **1W** | *3* | *44* | *45* | *45* | *45* | *6,67* | *97,78* | *100* | *100* |
|  | **SC** | **2** | *31* | *59* | *60* | *60* | *60* | *51,67* | *98,33* | *100* | *100* |
|  | **SC** | **4** | *0* | *49* | *49* | *49* | *51* | *0* | *96,08* | *96,08* | *96,08* |
|  | **SC** | **6** | *41* | *65* | *65* | *65* | *65* | *63,08* | *100* | *100* | *100* |
|  | **SC** | **9** | *0* | *50* | *51* | *51* | *54* | *0* | *92,59* | *94,44* | *94,44* |
|  | **SC** | **11** | *4* | *32* | *45* | *59* | *83* | *4,82* | *38,55* | *54,22* | *71,08* |
|  | **SPC** | **1W** | *1* | *62* | *63* | *63* | *63* | *1,59* | *98,41* | *100* | *100* |
|  | **SPC** | **2** | *2* | *71* | *71* | *71* | *71* | *2,82* | *100* | *100* | *100* |
|  | **SPC** | **4** | *57* | *85* | *86* | *86* | *86* | *66,28* | *98,84* | *100* | *100* |
|  | **SPC** | **6** | *20* | *63* | *71* | *75* | *81* | *24,69* | *77,78* | *87,65* | *92* |
|  | **SPC** | **9** | *2* | *52* | *54* | *59* | *60* | *3,33* | *86,67* | *90* | *98,33* |
|  | **SPC** | **11** | *5* | *29* | *35* | *40* | *83* | *6,02* | *35* | *42* | *48,19* |

1W = 1 week after ; SM = smooth mud, SC = smooth cement ; SPC = smooth paint cement ; WP 56.25 : mixture Clothianidin 200 mg m.a/m² + Deltamethrin 25 mg m.a/m² ; WG 250 : Deltamethrin 25 mg m.a/m² et WG70 : Clothianidin 200 mg m.a/m².
